# Inactivation of *Salmonella* spp., *Escherichia coli* O157:H7 and *Listeria monocytogenes* in Tahini by Microwave Heating

**DOI:** 10.3390/foods10122972

**Published:** 2021-12-02

**Authors:** Tareq M. Osaili, Anas A. Al-Nabulsi, Yasmeen M. Al Sheikh, Akram R. Alaboudi, Amin N. Olaimat, Murad Al-Holy, Walid M. Al-Rousan, Richard Holley

**Affiliations:** 1 Department of Clinical Nutrition and Dietetics, College of Health Sciences, The University of Sharjah, Sharjah P.O. Box 27272, United Arab Emirates; 2 Department of Nutrition and Food Technology, Faculty of Agriculture, Jordan University of Science and Technology, P.O. Box 3030, Irbid 22110, Jordan; anas_nabulsi@just.edu.jo (A.A.A.-N.); yasmeen_1993@yahoo.com (Y.M.A.S.); 3 Department of Pathology and Public Health, Faculty of Veterinary Medicine, Jordan University of Science and Technology, P.O. Box 3030, Irbid 22110, Jordan; akramalaboudi@yahoo.com; 4 Department of Clinical Nutrition and Dietetics, Faculty of Applied Medical Sciences, The Hashemite University, P.O. Box 330127, Zarqa 13133, Jordan; aminolaimat@hu.edu.jo (A.N.O.); murad@hu.edu.jo (M.A.-H.); 5 Department of Nutrition and Food Processing, Al-Huson College, Al-Balqa Applied University, P.O. Box 50, Al-Huson 21510, Jordan; walmm_53@yahoo.com; 6 Department of Food Science and Human Nutrition, University of Manitoba, Winnipeg, MB R3T 2N2, Canada; rick_holley@umanitoba.ca

**Keywords:** microwave, sesame paste, desiccation, starvation, D-value

## Abstract

Tahini (sesame paste) is a traditional food. Numerous foodborne outbreaks have been associated with it. This study aimed to (i) explore the efficiency of 2450 MHz microwave heating at 220, 330, 440, 550, and 660 W on the inactivation of *Salmonella* spp, *Escherichia coli* O157:H7, and *Listeria monocytogenes* in tahini; (ii) determine the impact of desiccation and starvation stresses on pathogen survival; (iii) assess the impact of microwave heating on the physicochemical characteristics of tahini. The inoculated microorganisms in tahini were reduced with higher microwave power levels (*p* < 0.05) and longer exposure times. The D-values of unstressed *Salmonella* spp., *Escherichia coli* O157:H7, and *L. monocytogenes* ranged from 6.18 to 0.50 min, 6.08 to 0.50 min, and 4.69 to 0.48 min, respectively, at power levels of 220 to 660 W, with z-values of 410, 440, and 460 W, respectively. Generally, desiccation and starvation stress levels prior to heating increased microbial resistance to heat treatment. Microwave heating did not affect acid, peroxide, *p*-anisidine, or color values of tahini up to 90 °C. These findings reveal microwave heating as a potential method for lowering the risk of *Salmonella* spp., *E. coli* O157:H7 and *L. monocytogenes* in tahini with no compromise on quality.

## 1. Introduction

Tahini (sesame paste) is a traditional food that is used as the main component in numerous dishes such as mutabbel, hummus, baba ghanoush, tarator sauce, salad dressing, and halva in different countries of the Middle East (Levant region and Gulf area) [1,2,3,4]. Tahini is characterized as a high-fat (57–65% wt.), low-water-activity (a_w_) (0.16 to 0.25) food product [5]. Tahini has been involved in multiple foodborne-related outbreaks and product recalls due to microbial contamination, including *Salmonella* and *L. monocytogenes* [6]. Tahini samples were also reported to be contaminated with coliforms [7] and *E. coli* [8].

Tahini preparation begins with the cleaning of sesame seeds by rinsing in water to remove any foodborne pathogens that might be present from foreign material. After that, caustic soda solution and mechanical friction are applied. Then, the seeds are dehydrated mechanically by a current of hot air, and then, they are dehulled, roasted, and milled to produce tahini [2,9]. Sesame seed is usually roasted at 110–150 °C for 30–60 min, and this is usually deemed adequate to destroy vegetative foodborne pathogens such as *Salmonella* [10]. Yet, *Salmonella* was found on sesame seeds after roasting despite low a_w_ [11]. Thus, the existence of microorganisms in tahini could be because they survived roasting [11]. The lethality of the roasting process on *Salmonella* present on the sesame seeds depends on the a_w_, with low a_w_ encouraging survival [11]. If *Salmonella* survives the roasting process, it is likely to remain viable throughout the shelf life [11]. However, the seeds may also become contaminated from the processing environment, workers, or equipment [5,6,10]. Additionally, *Salmonella* and *E. coli* O157:H7 survive well upon different storage conditions [12,13]. Therefore, some form of intervention that eliminates foodborne pathogens in this product without a compromise on sensory properties is needed. The use of microwave heating has increased dramatically for home cooking and commercial processing of foods [14] because of its capability to achieve high heating rates and lower the cooking time, with little change in organoleptic properties and nutritional values, commonly associated with other heating techniques [15]. Microwave frequencies commonly used are 2450 MHz (domestic microwave ovens) and 915 MHz (industrial equipment), respectively [16]. Microwave treatment may be especially useful in tahini, compared with other thermal treatments, because microbial contamination may occur during the later stages of production [11]. A microwave treatment can be given to the product after packaging. This will ensure the microbiological safety of the product.

Microwave heating was used to inactivate *Salmonella* in raw poultry, popcorn, corn-soy milk, frozen food, and rice salad [17]. It was also used to inactivate *S. Typhimurium*, *E. coli* O157:H7, and *L. monocytogenes* in salsa and peanut butter [18,19,20]. A 915 MHz microwave oven treatment at 2, 4, and 6 kW for peanut butter for 1, 3, and 5 min was also performed [19]. The reduction in *Salmonella* spp. increased as the time and power increased. Samples treated at 6 kW for 5 min had *Salmonella* spp. reduced by 3.2–4.3 log_10_ CFU/g. A 2 and 4 kW treatment for 5 min reduced *Salmonella* spp. by 0.2–0.4 and 1.1–1.5 log_10_ CFU/g, respectively. For acid and peroxide values, both indices increased significantly, compared with the control samples. However, the color did not change (*p* < 0.05) as the power of the microwave increased to 6 kW. Some other studies have induced pathogen stress in tahini by decreasing pH or by the use of essential oils [21,22].

Environmental stresses such as desiccation and starvation prior to heat challenge can significantly increase the thermal resistance of microorganisms [23]. Desiccating bacterial cells decreases intracellular molecular mobility and stabilizes the ribosomes upon the application of thermal treatment, preventing extensive harm in low-moisture environments [24]. Starvation stress causes cells to become more resistant to heat and oxidation challenges by induction of new protective proteins [25].

To the best of our knowledge, no studies have evaluated the effectiveness of microwave heating in eliminating foodborne pathogens from tahini. Therefore, the aims of this work were to (i) explore the efficiency of 2450 MHz microwave heating at 220, 330, 440, 550, and 660 W on the inactivation of *Salmonella* spp., *Escherichia coli* O157:H7, and *Listeria monocytogenes* in tahini; (ii) determine the impact of desiccation and starvation stresses on pathogen survival; (iii) assess the impact of microwave heating on the physicochemical characteristics of tahini.

## 2. Materials and Methods

### 2.1. Bacterial Culture Preparation

Four serovars of *Salmonella* (*S. Typhimurium* T231, *S. Typhimurium* T123, *S. Cubana* T109, *S*. *Aberdeen* T069) were isolated locally from tahini or tahini halva, 4 strains of *L. monocytogenes* isolated from local food items (foul, falafel, salad, and mashed potato) and 4 strains of verotoxin negative *E. coli* O157:H7 (Agriculture and Agri-Food Canada, Food Research Institute, Ottawa, and Health Canada, Microbial Hazards Laboratory, Ottawa, Canada) were used in this study [26]. Bacterial cultures were prepared as described in a previous study [27]. The bacterial cultures were stored at −40 °C in Brain Heart Infusion (BHI, Oxoid Ltd., Basingstoke, UK) Broth with 20% glycerol. A loopful of each culture was inoculated in tryptic soy broth (TSB, Oxoid). The mixture was incubated at 37 °C for 24 h. To reinvigorate the bacteria, three sequential transfers were performed. A cocktail of each bacterium was prepared by mixing 2.5 mL of each strain culture and then centrifuged (4000× *g*; 15 min). The pellet was resuspended in 1 mL sterile peptone water (Oxoid). The final concentration of the prepared cultures was tested on selective media and found to be 10^8^–10^9^ CFU/mL.

### 2.2. Stressed Bacterial Suspension Preparation

Desiccation and starvation stress conditions were established as per published protocols [13,27].

#### 2.2.1. Desiccation Stress

Desiccation stress was achieved by dividing 1 mL of each fresh culture into micro portions of 25 μL in a sterile Petri dish that was kept uncovered at room temperature (21 ± 1 °C) for 2 h in a biosafety cabinet. The desiccated cells were then amassed by pouring 10 mL of potassium phosphate buffer (PPB) into the plate. The plate was shaken by hand several times to encourage cell assembly [27].

#### 2.2.2. Starvation

Starvation stress was achieved by adding 1 mL of each fresh culture to a 9 mL sterile saline solution (0.85% NaCl) that was vortexed well and incubated at 37 °C for 48 h [13].

### 2.3. Tahini Samples and Bacterial Inoculation

Fresh tahini was obtained from local markets. *Salmonella*, *E. coli* O157:H7, and *L. monocytogenes* were not found in Tahini samples before use [28,29,30]. Tahini samples (100 g) were inoculated with 1 mL of unstressed or stressed cocktail cultures and mixed thoroughly with a sterile spatula to distribute the culture consistently through the sample. The inoculation level of the samples was tested several times by plating the diluted samples on selective media and was found to be 10^6^–10^7^ CFU/g. Then, 25 g of inoculated sample were placed in a sterile 250 mL beaker for microwave treatment.

### 2.4. Microwave Heating

The samples (25 g) were exposed to microwave radiation using a microwave oven (model EM45LWGK, 45 l, Sona, Jordan) at 2450 MHz. The samples were placed inside the oven cavity in the center of the turntable and exposed to 5 different power levels for different intervals (220 W for 7, 14, 21, and 28 min; 330 W for 2, 4, 6, and 8 min; 440 W for 1, 2, 3, and 4 min; 550 W for 40, 80, 120, and 160 s; and 660 W for 30, 60, 90, and 120 s). This was carried out to understand the role temperature played in association with power level on bacterial destruction. The temperature of uninoculated samples during microwave heating was measured by a digital thermometer (ThermoPro, Toronto, ON, Canada).

### 2.5. Bacterial Enumeration

A 5 g sample was added to 45 mL of sterile 0.1% peptone water (Oxoid, UK) and treated in a stomacher (Seward, Ltd., London, UK) for 2 min. Samples were then decimally diluted (0.1% peptone water) and plated on xylose lysine deoxycholate agar for *Salmonella*, sorbitol MacConkey agar with a cefixime tellurite selective supplement for *E. coli* O157:H7, and Oxford agar with modified Listeria selective supplement for *L. monocytogenes* overlaid with tryptic soy agar (TSA, Oxoid) for injured cell recovery [27].

### 2.6. D-Value Calculation

Microsoft Excel (Microsoft, Redmond, Washington, DC, USA) was used to calculate D-value (time required to inactivate 1 log_10_ of the bacterial population at a specific power level) and z-value (the number of watts the power would need to be increased by to achieve a tenfold (i.e., 1 log_10_) reduction in the D-value).

Common logarithms of microbial survival in tahini after microwave heating were plotted against the time intervals for each power level used. The D-value was calculated using a linear regression model as follows:

Log_10_ (N) = Log_10_ (N_0_) − r/D (1)

where

r = Time (min);

N = Number of survivors (CFU/g) at r;

N_0_ = Number of survivors (CFU/g) at time 0;

D = The decimal reduction time (min) at specific microwave power.

Goodness of fit was determined from the R^2^ (coefficient of determination). Models with R^2^ ≥ 0.9 were considered a good fit of data.

The z-value was calculated using a linear regression model as follows:

Log_10_ D = log_10_ D_0_ − P/z (2)

where D is the decimal reduction time (min) at power level P (W); D_0_ is the decimal reduction time at power 220 W; Z = power resistance constant (W); goodness of fit was determined from the R^2^. Models with R^2^ ≥0.9 were considered to have a good fit of data.

### 2.7. Chemical and Physical Analysis of Tahini Samples

#### 2.7.1. Proximate Analysis and a_w_

Carbohydrate, protein, fat, ash, and moisture content were determined on triplicate samples as per the American Association of Official and Oil Chemists (AOAC) protocol [31]. Protein and fat contents were determined by the Kjeldahl method and Soxhlet Apparatus, respectively. Ash was determined using a Carbolite furnace (CSF 1100, Germany). Moisture was determined by drying the sample to obtain a constant weight. Carbohydrate was determined as the difference. The a_w_ was determined for a 5 g sample at 21 °C using a water activity meter (Hygrolab, Rotronic Instr. Crop, New York, NY, USA) [32].

#### 2.7.2. Acid Value, Peroxide Value, and *p*-Anisidine Value Determination

The methods described by AOAC were followed to determine the peroxide value and acid value (mg Potassium Hydroxide (KOH) per g sample) for both control and treated tahini [33]. The acid value was calculated as per the following equation:

(3) $$\mathrm{acid}\;\mathrm{value}\;\left(\mathrm{mg}\;\mathrm{KOH}\;\mathrm{per}\;\mathrm{gram}\right)=\frac{\mathrm{titre}\;\mathrm{value}*\mathrm{normality}\;\mathrm{of}\;\mathrm{KOH}*56.1}{\mathrm{sample}\;\mathrm{weight}}$$

The method used to determine *p*-anisidine has also been published previously [34]. The average of three measurements was used to calculate each data point.

(4) $$\mathrm{peroxide}\;\mathrm{value}\;=\left[\frac{\mathrm{volume}\;\mathrm{of}\;\mathrm{sodium}\;\mathrm{thiosulphate}\;\left(\mathrm{ml}\right)*\mathrm{sodium}\;\mathrm{thiosulphate}\;\mathrm{normality}}{\mathrm{tahini}\;\mathrm{weight}\;\left(g\right)}\right]*100$$

#### 2.7.3. Color Measurement

The color of a 15 g sample was measured at three different sample spots in triplicate using Hunter colorimeter (Color TEC-PCMTM, Cole-Parameter International, Accuracy Microsensors, Inc. Pittsford, NY, USA) [27]. Observations were expressed as L *, a *, and b * representing lightness, redness, and yellowness of the sample.

### 2.8. Statistical Analysis

The experiments were repeated in triplicate and the statistical analysis was performed using SPSS software version 19.0 (2009; Chicago, IL, USA). The average D-values of stressed cells were compared with those of unstressed cells using Student’s *t*-test. One-way ANOVA was used to examine the effect of microwave heating on quality parameters with respect to microwave power. Tukey’s test was conducted to determine the difference among variable groups. A *p*-value of <0.05 was considered to be significant.

## 3. Results

### 3.1. Microwave Heating Impact on Stressed and Unstressed Salmonella spp.

**Figure 1:**
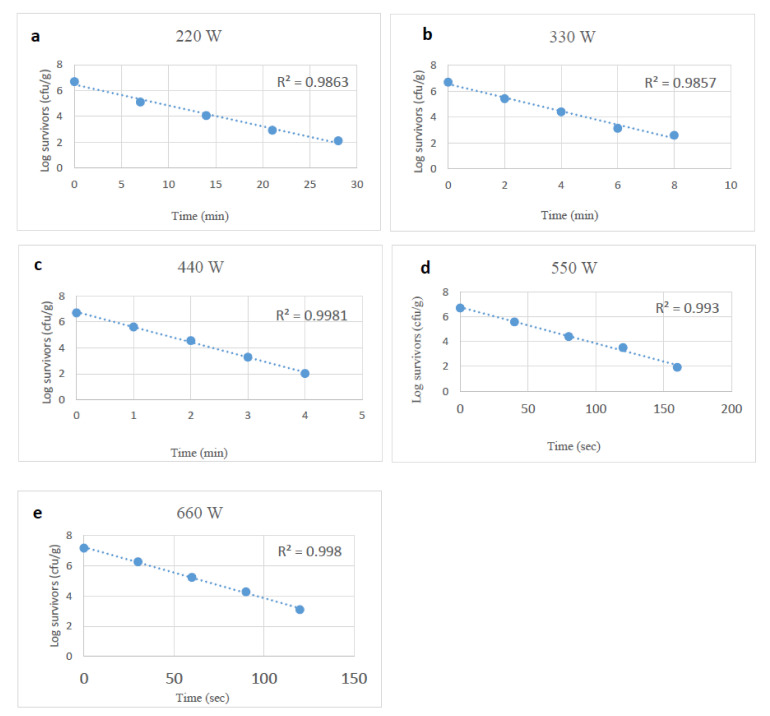
Survival curves of unstressed (control) *Salmonella* spp., after exposure to microwave heating at 220, 330, 440, 550 and 660 W (**a**–**e**).

**Figure 2:**
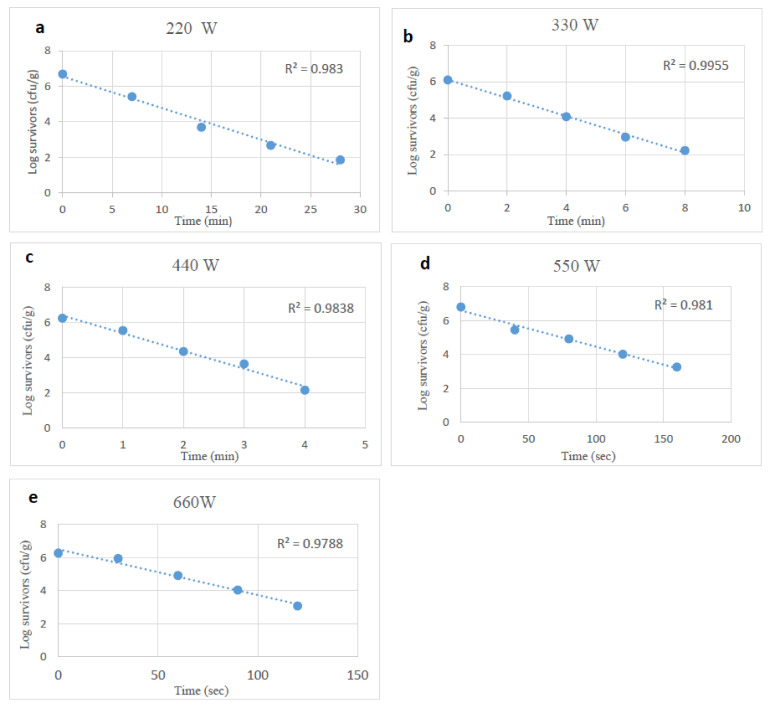
Survival curves of desiccation stressed *Salmonella* spp., after exposure to microwave heating at 220, 330, 440, 550 and 660 W (**a**–**e**).

**Figure 3:**
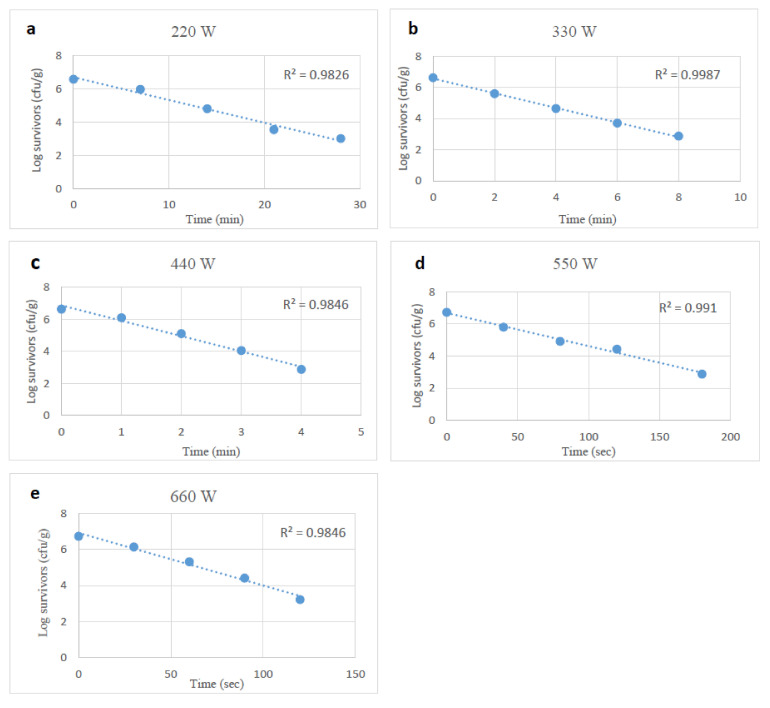
Survival curves of starvation stressed *Salmonella* spp., after exposure to microwave heating at 220, 330, 440, 550 and 660 W (**a**–**e**).

Survivor curves of desiccation and starvation-stressed plus unstressed or control *Salmonella* spp. in tahini after exposure to microwave heating at 220, 330, 440, 550, and 660 W were generated by fitting the surviving cells (log_10_) and microwave heating time in a linear regression model. The R^2^ value was >0.98 (Figure 1, Figure 2 and Figure 3). The counts of the microbial cells grown on the selective media overlaid with TSA were used to calculate the D-values of unstressed and stressed cells. At each power level, the survival of *Salmonella* cells decreased linearly as exposure time to microwaves increased. Microwave heating at 220 to 660 W reduced unstressed *Salmonella* spp. numbers by 0.5 to 4.8 log_10_ CFU/g at treatment conditions varying from 160 sec to 28 min (based on the microwave power level). D-values of *Salmonella* spp. decreased (*p* < 0.05) as microwave power levels increased. The D-values of unstressed cells at 220 to 660 W ranged from 6.18 to 0.50 min (Table 1).

The extent of reductions in the viability of desiccation-stressed *Salmonella* by microwave heating was similar to those of unstressed cells at the same power levels, but effects occurred more slowly. The D-values of desiccation-stressed *Salmonella* at power levels of 220 to 660 W min ranged from 5.64 to 0.60 min. The D-values at 440, 550 and 660 W were higher (*p* < 0.05) than those of unstressed *Salmonella* cells (Table 1).

With starvation-stressed *Salmonella*, again, reductions following similar microwave treatments ranged from 0.6 to 4.0 log_10_ CFU/g, and reductions occurred more slowly than with unstressed cells. The D-values of starvation-stressed *Salmonella* spp. at 220 to 660 W ranged from 7.29 to 0.57 min, and were higher (*p* < 0.05) than those of unstressed *Salmonella* cells (Table 1).

### 3.2. Microwave Heating Impact on Stressed and Unstressed E. coli O157:H7

**Figure 4:**
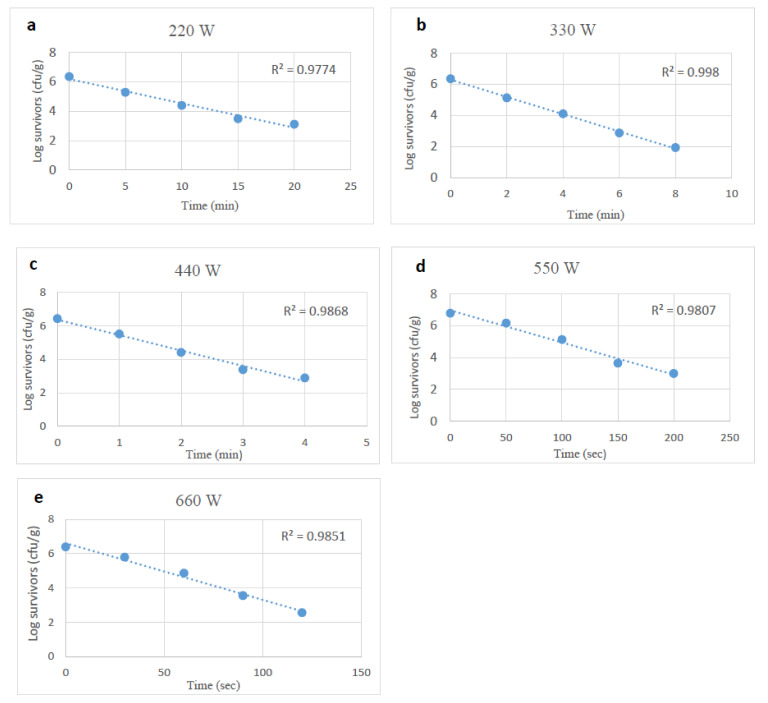
Survival curves of unstressed (control) *E. coli O157:H7* after exposure to microwave heating at 220, 330, 440, 550 and 660 W (**a**–**e**).

**Figure 5:**
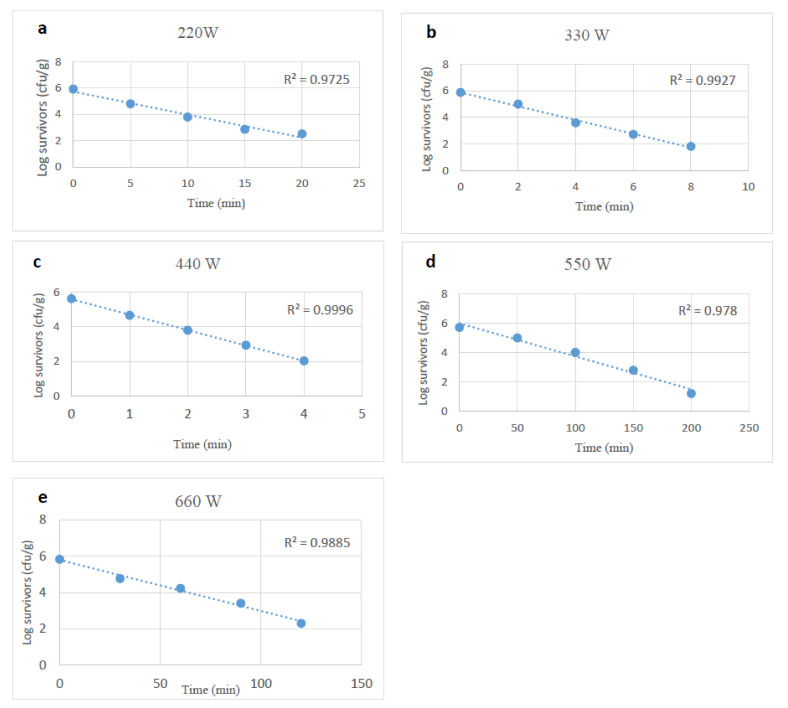
Survival curves of desiccation stressed *E. coli O157:H7* after exposure to microwave heating at 220, 330, 440, 550 and 660 W (**a**–**e**).

**Figure 6:**
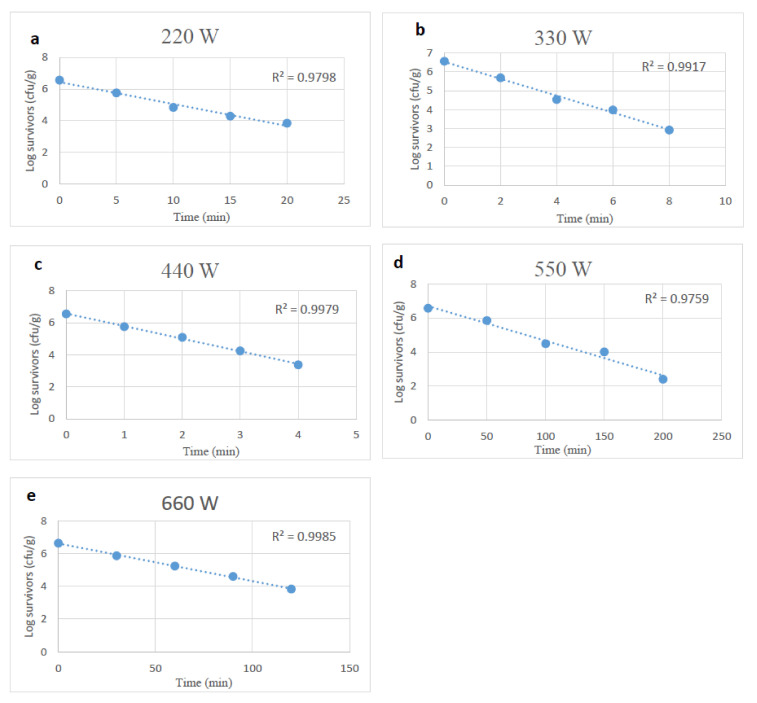
Survival curves of starvation stressed *E. coli O157:H7* after exposure to microwave heating at 220, 330, 440, 550 and 660 W (**a**–**e**).

*E. coli* O157:H7 survival decreased linearly as microwave exposure increased (Figure 4, Figure 5 and Figure 6). Heating at 220 to 660 W reduced *E. coli* O157:H7 by 0.3 to 4.5 log_10_ CFU/g following treatments lasting from 50 s to 20 min. The D-values at power levels of 220 to 660 W ranged from 6.08 to 0.50 min (Table 2).

Microwave heating at 220 to 660 W reduced desiccation-stressed *E. coli* O157:H7 by 0.8 to 4.6 log_10_ CFU/g following treatments that again lasted from 50 s to 20 min. The D-values of desiccation stressed *E. coli* O157:H7 at 220 to 660 W ranged from 5.76 to 0.60 min. The D-values at power levels of 550 and 660 W were significantly higher than the D-values of unstressed *E. coli* O157:H7 (Table 2).

Microwave heating at 220 to 660 W reduced starvation-stressed *E. coli* O157:H7 by 0.7 to 4.2 log_10_ CFU/g during treatments that lasted 30 s to 20 min. The D-values of starvation-stressed *E. coli* O157:H7 at power levels of 220 to 660 W ranged from 6.91 to 0.60 min, and these values were not significantly different from those of unstressed cells. (Table 2).

### 3.3. Microwave Heating Impact on Stressed and Unstressed Listeria Monocytogenes

**Figure 7:**
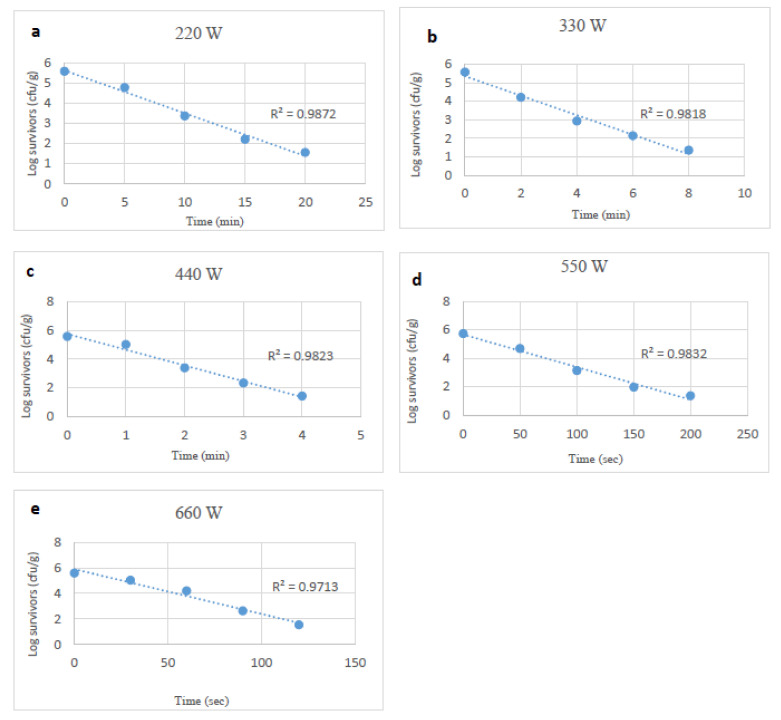
Survival curves of unstressed (control) *L. monocytogenes* after exposure to microwave heating at 220, 330, 440, 550 and 660 W (**a**–**e**).

**Figure 8:**
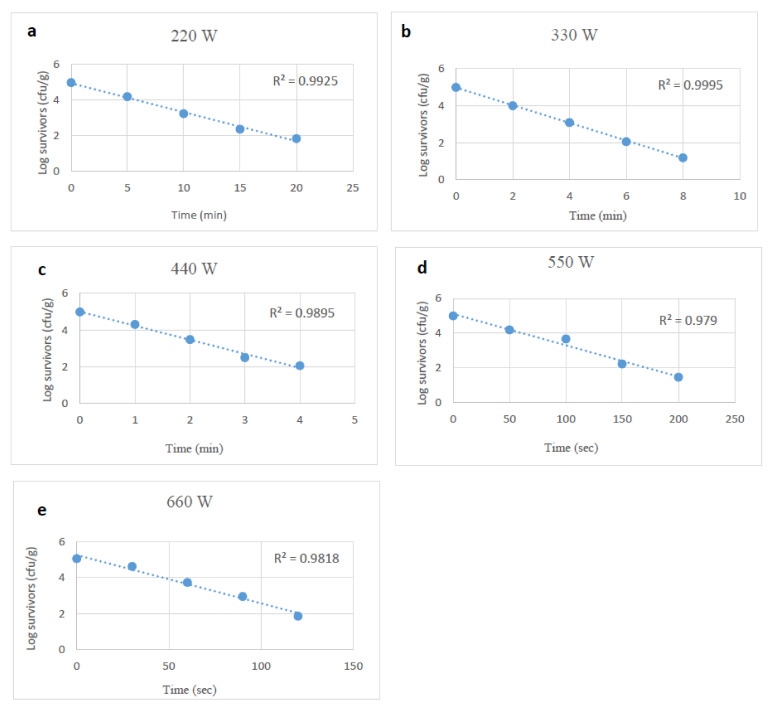
Survival curves of desiccation stressed *L. monocytogenes* after exposure to microwave heating at 220, 330, 440, 550 and 660 W (**a**–**e**).

**Figure 9:**
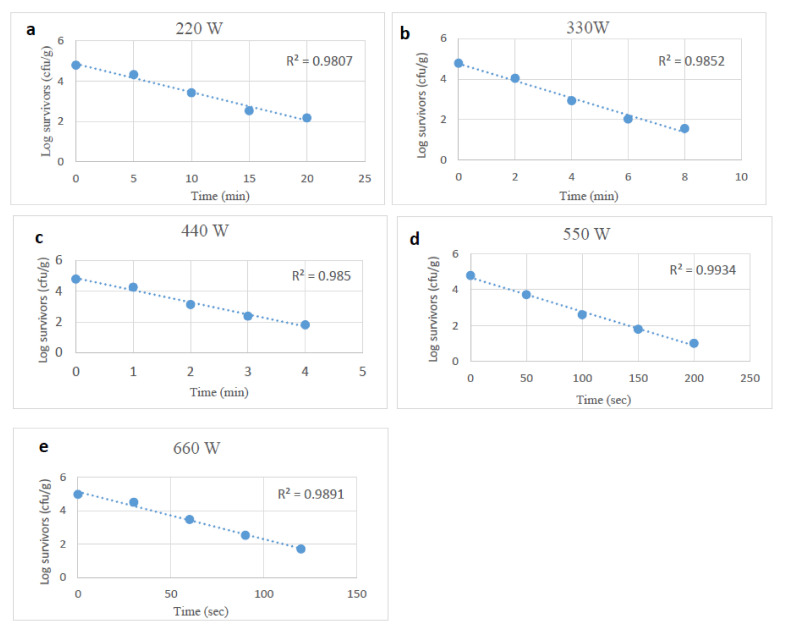
Survival curves of starvation stressed *L. monocytogenes* after exposure to microwave heating at 220, 330, 440, 550 and 660 W (**a**–**e**).

At each power level, the survival of *L. monocytogenes* decreased linearly as the exposure time to the microwaves increased (Figure 7, Figure 8 and Figure 9). Microwave heating at 220 to 660 W reduced *L. monocytogenes* viability by 0.6 to 4.3 log_10_ CFU/g over 50 s to 20 min. The D-values at power levels of 220 to 660 W ranged from 4.69 to 0.48 min (Table 3).

The treatment of desiccation-stressed *L. monocytogenes* by 220 to 660 W microwave power caused reductions of 0.4 to 3.6 log_10_ CFU/g following exposures lasting from 30 s to 20 min. The calculated D-values of desiccation-stressed *L. monocytogenes* at 220 to 660 W ranged from 6.17 to 0.63 min. These were significantly (*p* < 0.05) higher than those of unstressed *L. monocytogenes* (Table 3).

Treatment of starvation-stressed *L. monocytogenes* by 220 to 660 W microwave power reduced organism viability by 0.3 to 3.3 log_10_ CFU/g over 50 s to 20 min. The D-values of starvation-stressed *L. monocytogenes* at 220 to 660 W ranged from 7.15 to 0.59 min. The D-values of starvation-stressed *L. monocytogenes* were significantly higher than those of unstressed *L. monocytogenes* (Table 3).

### 3.4. Stress-Induced Changes in z-Values

With *Salmonella*, the z-values of desiccation-stressed cells were significantly higher (*p* < 0.05) than that of the unstressed control (Table 4). Similarly, with *E. coli* O157:H7, the z-values of both types of stressed cells were higher than that of the unstressed control (Table 4). In contrast, with *L. monocytogenes*, the z-values of both the control and desiccation-stressed cells were similar, while starvation-stressed cells showed a z-value that was significantly lower than that of unstressed cells (Table 4).

### 3.5. Thermal Profile of Tahini during Microwave Heating at Different Power Levels

**Figure 10:**
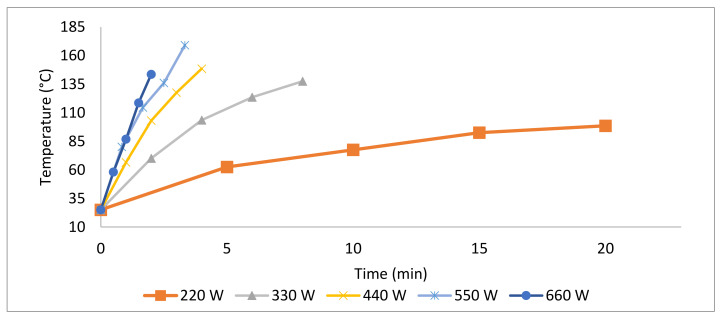
Time–temperature profile of tahini during microwave heating at different power levels.

As per Figure 10, the temperature increased at 220, 330, 440, 550, and 660 W from 23 °C to 99 ± 2.1, 138 ± 0.7, 149 ± 2.1, 169 ± 29.7, and 144 ± 0.7 °C within 20 min, 8 min, 4 min, 3.3 min, and 2 min, respectively.

### 3.6. Proximate Composition and a_w_ of Tahini

Tahini contained 57.6 ± 0.1% total fat, 27.91 ± 0.04% crude protein, 9.34 ± 0.80% carbohydrate, 3.06 ± 0.05% ash, 0.1 ± 0.01% moisture, and the a_w_ was 0.16 ± 0.01.

### 3.7. Color Analysis of Control and Microwave-Treated Tahini

As can be seen in Table 5, the values of lightness (L *) and yellowness (b *) of heated and non-heated samples were similar (*p* ≥ 0.05) at temperatures up to 120 °C. A similar effect was seen in redness (a *) of samples heated up to 90 °C.

### 3.8. Acid, Peroxide and p-Anisidine Values of Microwave-Heated Tahini

Acid values of the tahini samples heated to temperatures of 23, 60, 90, 120, and 150 °C using a power level of 440 W ranged from 0.82–1.00 mg KOH/g, with no significant differences between the control and samples that reached 60 °C, but the values increased (*p* < 0.05) in samples that reached temperatures of 90, 120, and 150 °C (Table 6). The calculated peroxide values ranged from 1.52 to 1.65 meq O_2_/kg, and the values in samples that reached 120 °C were not significantly different; however, the peroxide value slightly increased (*p* < 0.05) in samples that reached 150 °C. The *p*-anisidine values for both control and microwave-heated tahini samples ranged from 1.64 to 2.80 and values increased significantly as temperature increased during microwave treatment.

## 4. Discussion

Previous reports have shown that *Salmonella* can survive in tahini and its byproduct (halva) for up to a year [32,35]. Similarly, *L. monocytogenes* and *E. coli* O157:H7 were reported to grow well in tahini despite it being stored at 10 °C [36]. About 43% of tahini samples were found to be contaminated with *E. coli* in Lebanon [37]. Thus, a feasible method that would decrease the viability of these pathogens would be of significant value.

In this study, microbial populations were reduced by increasing microwave heating time and power level. The same trend was observed previously in different food products [17,19,38]. Microwaves inactivate microorganisms by dielectric heating where electromagnetic radiation penetrates the cytoplasm and generates heat within it by interaction with water molecules [39]. This can lead to irreversible thermal denaturation of enzymes, proteins, nucleic acids, and eventually cell death. Other proposed mechanisms include “electroporation”, where changes in cell membrane electrical potential generate pores, resulting in leakage of cellular materials. In addition, the magnetic field of the microwave can destroy protein and DNA, which are essential for pathogen survival [40]. In the current study, the temperature of tahini during heating with a 2450 MHz microwave oven was measured at 5 power levels. After 2 min, the temperatures in the center of tahini samples reached 45, 70, 103, 119, and 144 °C at 220, 330, 440, 550, and 660 W, respectively. A similar pattern was noted in peanut butter [19], in which an increase of up to 22, 49, and 101 °C at 2, 4, and 6 kW (after 5 min of microwave heating) occurred, respectively. After 50 s of heating at 700 W using a household microwave oven, more than a 2.1 log_10_ reduction in *S. enterica* was achieved in grape tomatoes, while a >1.7 log_10_ reduction was detected after 50 s treatment at 350 W [41]. In this study, it was found that microwave heating at 220, 330, 440, 550, and 660 W inactivated unstressed *Salmonella* spp., *E. coli* O157:H7 and *L. monocytogenes* in Tahini by 3.6–4.7, 3.3–4.5 and 4.1–4.3 log_10_ CFU/g, respectively. Microwaves (915 MHz; 1.2–4.8 kW) caused reductions in *S.* Typhimurium, *E. coli* O157:H7, and *L. monocytogenes* in salsa by 5.8–6.1, 5.2–6.2, and 4.5–4.8 log_10_ CFU/g, respectively [18]. In peanut butter, microwave heating (915 MHz, 5 min) at 6, 4, and 2 kW reduced the viability of *Salmonella* serovars by 3.2–4.3, 1.1–1.5, and 0.2–0.4 log_10_ CFU/g, respectively [19]. Microwave effects upon microorganisms depend on many factors such as machine capability, food dielectric properties and characteristics of the target microorganisms as well as the level of contamination, plus the physiological state and growth phase of the microorganism [42,43,44]. Moreover, the water content and a_w_ of the product can affect the sensitivity of the test organism towards microwave heating. For example, salsa has higher water and lower fat content, compared with tahini, which has an a_w_ of 0.16, or that of peanut butter (a_w_ = 0.26), and consequently, salsa showed the greatest microbial reduction during microwave heating. Additionally, the presence of oil in tahini and peanut butter decreases the heating capacity of microwaves because fat enables only low molecular dipole movement [45]. Salt in salsa tends to increase the rate of heating due to its dielectric properties. Both sample and container size can affect the rate of temperature increase within the sample [46]; larger food masses take longer to be heated, compared with smaller masses [47].

In the current study, the D-values decreased significantly with increasing power levels. A similar trend was observed in previously conducted studies [19,48]. The D-value of *E. coli* in apple juice at 270 W was 3.88 min while at 900 W it was 0.42 min [48]. The T_d_-values (used when survivor curves show shoulders) for *Salmonella* Typhimurium, *E. coli* O157:H7, and *L. monocytogenes* in peanut butter with an a_w_ of 0.3 at 4 and 6 kW (915 MHz) were 4.75 and 2.56, 4.59 and 2.17, and 17.28 and 3.34 min, respectively [20]. Furthermore, the authors stated that the T_d_ values of the tested microorganisms were similar to the D-values of *Salmonella* exposed to conventional heating at 90 °C. Similar to the present findings, a 1 log_10_ reduction in *E. coli* O157:H7 after 35 s of microwave heating at 652 W in apple purée has been reported [49]. However, the authors observed a 7 log_10_ reduction in *L. innocua* using the same power and time. The D-values of *L. monocytogenes* in a kiwi fruit purée at 600, 900 and 1000 W (60 °C) were 42.85, 17.35, and 17.04 s, respectively [38]. Another factor that may affect microwave inactivation of pathogens is the holding time after microwave treatment. A 2 min holding period after microwave treatment completely inactivated *L. monocytogenes* that had been inoculated on shrimp [50]. In the current study, there was no holding time; the samples were combined with peptone water immediately after the treatment to interrupt the effect of heat. Another reason for the log_10_ reduction differences between the present and the previous studies is the microwave frequency. Higher temperatures were observed in beef samples exposed to microwave radiation at 915 MHz, compared with 2450 MHz [46]. Microwave ovens with lower frequencies resulted in a more rapid increase in temperature and greater depth of energy penetration in food samples, compared with those with higher frequencies [46]. It was found that *E. coli* O157:H7 and *L. monocytogenes* had higher z-values than *Salmonella* spp. In contrast, z-values of 6.2 (*Salmonella*), 5.4 (*E. coli* O157:H7), and 5.9 °C (*L. monocytogenes*), respectively, were observed in meat samples heated in a water bath [51]. D- and z-values depend on the strain, medium, or food and treatment conditions [52]. Moreover, microbial destruction is faster under microwave than conventional heating. The z-value of *Saccharomyces cerevisiae* and *Lactobacillus plantarum* in apple juice was lower in the microwave (7, 4.5 °C), compared with conventional heating (13.4, 15.9 °C) [53]. *L. monocytogenes* counts in tahini upon exposure to high pressure (600 MPa for 600 s) did not significantly decrease [54]. Thus, using a microwave as a method to inactivate pathogenic microorganisms could be considered promising and a feasible option. However, one important limitation in microwave heating is the non-uniform temperature distribution that occurs in food products, which is likely caused by differences in the thermal and dielectric properties of their constituents. This phenomenon leads to formation of cold and hot spots in the product that can affect final product quality and safety and may lead to the survival of some pathogenic bacteria [15].

Extreme changes in environmental conditions from the optimum to sub-optimum create stress on an organism [55]. In this study, the effects of desiccation or starvation on the response of *Salmonella* spp., *E. coli* O157:H7, and *L. monocytogenes* in tahini to microwave heating were investigated. To the best of our knowledge, there are no data available that characterize the effect of stress on microorganism sensitivity toward microwave heating in tahini.

Desiccation stress significantly affected the inactivation of *Salmonella* spp. and *L. monocytogenes* in tahini by increasing their heat resistance, as indicated by increased D-values at most microwave power levels. In contrast, desiccation caused significant reductions in the D-values of *Cronobacter* spp. cells in infant milk formula [56]. In addition, *Cronobacter* spp. were more susceptible to thermal inactivation after storage in the formula [57]. It has been observed that *Salmonella* showed significantly greater resistance to desiccation than *L. monocytogenes* and *E. coli* O157:H7 [58]. In this study, starvation stress cross-protected *L. monocytogenes*, *Salmonella* spp., and increased their thermal resistance significantly. Starvation stress increased the thermotolerance of microorganisms in food [59,60]. *L. monocytogenes* Scott A that had undergone starvation stress (48 h) had an increased D-value by 5.5-fold [60]. However, the thermal resistance of starvation-stressed *E. coli* O157:H7 cells did not change significantly, compared with unstressed *E. coli* O157:H7 cells, except at 660 W. This latter difference might have been due to variations in thermal resistance of the microbial strains used in that study. Similar results were obtained previously, indicating that starvation stress did not affect thermal resistance of *E. coli* O157:H7 [61]. The authors explained that reaching an internal temperature of 65 °C was adequate to overcome resistance triggered due to expression of shock proteins.

Survival of bacteria after exposure to stress from compatible solutes, biofilms, and dormancy could be due to different mechanisms [62,63,64]. Microbial cell resistance could either be stress specific or as a result of general under/overexpression of genes catering to stress response; the common regulators include sigma factors, phosphorelay-based two-component systems or may be transcription related [65]. Pathogenic microorganisms require water to sustain functionality, but desiccation restricts this availability and hence intensifies bacterial competition. Osmotic dehydration (desiccation) results in the formation of trehalose; a compound that forms a protective envelope around proteins and other macromolecules, thereby helping the pathogen to maintain shape and avoid being denatured [66,67,68,69]. Scarcity of water due to desiccation inhibits protein hydration, which eventually leads to loss of functionality [69].

In this study, microwave heating did not impact tahini color (*p* < 0.05) up to 120 °C. Upon increasing the temperature to 150 °C, the lightness (L *) decreased from 57.87 to 57.00, redness (a *) increased from 1.07 to 1.54, and yellowness (b *) increased from 29.37 to 30.57. Similar findings were observed in peanut butter, in which after an increase from 0 to 6 kW (915 MHz), L * values decreased, and although both a * and b * increased slightly, these differences were not significant [19]. Additionally, no color parameters of grape tomatoes showed significant changes after microwave heating up to 48 °C [41]. However, microwave heating decreased lightness (L *) from 30.38 to 24.35 and 25.98 to 19.61 in jalapeno pepper and coriander, respectively [70]. Food color alterations during cooking could be attributed to the oxidation of ascorbic acid, pigment degradation, and browning [71]. Tahini was more resistant to color change, and this might be explained by the low a_w_ (0.16) of tahini samples and the presence of phenolic and antioxidant compounds, which have the ability to retard redox reactions, including the Millard reaction, leading to color changes [72].

The lipid oxidation indicators (acid, peroxide, and *p*-anisidine values), are considered key contributors to the quality decline in high lipid foods. Lipid oxidation can be stimulated by both thermal and non-thermal treatments. For example, pulsed electric field treatment increased peroxide values [73]. Similarly, irradiation did not affect acid values in Tahini halva samples treated with up to 4 kGy. However, samples treated with ≥1.6 and ≥2.4 kGy affected peroxide and *p*-anisidine values significantly [27]. In this study, acid, peroxide, and *p*-anisidine values at 23–150 °C ranged from 0.82 to 1.00 mg KOH/g, 1.5–1.65 meq O_2_/kg, and 1.64–2.80 *p*AV, respectively, with no significant increase, even at temperatures above 90 °C. Activation energy needed for lipid oxidation reduces upon temperature increase [74]. The present findings were comparable to those reported in peanut butter, in which acid and peroxide values were slightly higher after a microwave treatment (change from 1.04 to 1.12 mg KOH/g for acid and from 10.85 to 11.50 meq O_2_/kg for peroxide, respectively (*p* > 0.05)) [19]. In another study, higher peroxide and *p*-anisidine values after microwave roasting of sunflower oil were observed for 12 min, and clear differences (*p* < 0.05) were evident following 20 min of treatment [75]. At higher temperatures, a complex pattern of thermolytic and oxidative reactions occurred, which can lead to increases in acid, peroxide, and *p*-anisidine values [76].

## 5. Conclusions

To the best of our knowledge, this is the first study to indicate that microwave heating can be used to eliminate stressed and unstressed *Salmonella* spp., *E. coli* O157:H7, and *L. monocytogenes* in tahini without affecting its chemical and physical properties. As the contamination of tahini usually occurs during the later stages of production, this technique would be convenient for producers, as the treatment can be given even after packaging. This study may help tahini producers to validate the process lethality of the product.

## Figures and Tables

**Table 1:** D-values of unstressed and stressed *Salmonella* cocktail in tahini after exposure to microwave heating.

	D-Values (min)				
Watts	220	330	440	550	660
Control	6.18 ± 0.24 ^†,A^	1.91 ± 0.03 ^B^	0.86 ± 0.01 ^C^	0.57 ± 0.01 ^D^	0.50 ± 0.02 ^D^
Desiccation	5.64 ± 0.19 ^A,^*	2.01 ± 0.08 ^B^	0.99 ± 0.03 ^C,^*	0.79 ± 0.05 ^D,^*	0.60 ± 0.05 ^E,^*
Starvation	7.29 ± 0.04 ^A,^*	2.13 ± 0.09 ^B,^*	1.05 ± 0.04 ^C,^*	0.81 ± 0.02 ^D,^*	0.57 ± 0.02 ^E,^*

^†^ Values represent means of three replications ± standard deviation. Means in the same row with the same capital letter are not significantly different (*p* ≥ 0.05). * Means within the same column are significantly different (*p* < 0.05) from the control.

**Table 2:** D-values of unstressed and stressed *E. coli* O157:H7 cocktail in tahini after exposure to microwave heating.

	D-Values (min)				
Watts	220	330	440	550	660
Control	6.08 ± 0.54 ^†,^^A^	1.80 ± 0.20 ^B^	1.04 ± 0.11 ^C^	0.84 ± 0.03 ^C,D^	0.50 ± 0.01 ^D^
Desiccation	5.76 ± 0.54 ^A^	1.93 ± 0.10 ^B^	1.14 ± 0.12 ^C^	0.86 ± 0.12 ^C,D^	0.60 ± 0.05 ^D,^*
Starvation	6.91 ± 0.75 ^A^	1.93 ± 0.10 ^B^	1.28 ± 0.12 ^C^	0.83 ± 0.08 ^C,D^	0.60 ± 0.05 ^D,^*

^†^ Values represent means of three replications ± standard deviation. Means in the same row with the same capital letter are not significantly different (*p* ≥ 0.05).* Means within the same column are significantly different (*p* < 0.05) from the control.

**Table 3:** D-values of unstressed and stressed *L. monocytogenes* cocktail in tahini after exposure to microwave heating.

	D-Values (min)				
Watts	220	330	440	550	660
Control	4.69 ± 0.21 ^†,A^	1.93 ± 0.20 ^B^	0.91 ± 0.10 ^C^	0.72 ± 0.04 ^C,D^	0.48 ±0.02 ^D^
Desiccation	6.17 ± 0.48 ^A,^*	2.10 ± 0.08 ^B^	1.31± 0.07 ^C,^*	0.92± 0.01 ^C,D,^*	0.63 ± 0.04 ^D,^*
Starvation	7.15 ± 0.28 ^A,^*	2.38 ± 0.23 ^B^	1.28 ± 0.11 ^C,^*	0.88 ± 0.05 ^D,^*	0.59 ± 0.04 ^D,^*

^†^ Values represent means of three replications ± standard deviation. Means in the same row with the same capital letter are not significantly different (*p* ≥ 0.05). * Means within the same column are significantly different (*p* < 0.05) from the control.

**Table 4:** The z-values of stressed and unstressed *Salmonella* spp., *Escherichia coli* O157:H7, and *Listeria monocytogenes*.

Z-Values (Watts) ^†^			
Microorganism	Control	Desiccation	Starvation
Salmonella	410 † ± 9.6	470 ± 12.4 *	420 ± 10.0
Escherichia coli O157:H7	440 ± 18.9	480 ± 22.7	460 ± 12.5
Listeria monocytogenes	460 ± 12.5	470 ± 12.5	420 ± 10.5 *

^†^ Values represent means of three replications ± standard deviation.* Means within the same row are significantly different (*p* < 0.05) from the control.

**Table 5:** Color measurement ^†^ values of tahini samples at different temperatures generated by 440 W microwave treatment.

Temperature (°C)	L *	a *	b *
23	57.87 ± 0.32 ^††,A^	1.07 ± 0.07 ^A^	29.37 ± 0.13 ^A^
60	57.56 ± 0.07 ^A,B^	1.05 ± 0.06 ^A^	29.74 ± 0.21 ^A,B^
90	57.43 ± 0.04 ^A,B^	1.14 ± 0.08 ^A^	29.77 ± 0.93 ^A,B^
120	57.24 ± 0.08 ^A,B^	1.52 ± 0.40 ^B^	30.50 ± 0.09 ^A,B^
150	57.00 ± 0.92 ^B^	1.54 ± 0.13 ^B^	30.57 ± 0.42 ^B^

^†^ Color measurements: L * (lightness), a * (redness), and b * (yellowness). ^††^ Values represent means of three replications ± standard deviation. Means followed by the same capital letter within columns are not significantly different (*p* ≥ 0.05).

**Table 6:** Acid, peroxide, and *p*-anisidine values * of microwave heat-treated tahini samples.

Temperature (°C)	Acid Value (mg KOH/g)	Peroxide Value (meq O_2_/kg oil)	*p*-Anisidine Value
23	0.82 ± 0.01 ^†,^^A^	1.52 ± 0.02 ^A^	1.64 ± 0.01 ^A^
60	0.84 ± 0.01 ^A^	1.58 ± 0.02 ^A,B^	1.66 ± 0.01 ^A,B^
90	0.88 ± 0.02 ^B^	1.60 ± 0.05 ^A,B^	1.87 ± 0.09 ^B,C^
120	0.95 ± 0.01 ^C^	1.63 ± 0.05 ^A,B^	2.04 ± 0.12 ^C^
150	1.00 ± 0.01 ^D^	1.65 ± 0.07 ^B^	2.80 ± 0.11 ^D^

^†^ Values represent means of three replications ± standard deviation. Means followed by the same capital letters within columns are not significantly different (*p* ≥ 0.05).
